# Correction: Reaching adolescents with health services: Systematic development of an adolescent health check-ups and wellbeing programme in Ghana (Y-Check, Ghana)

**DOI:** 10.1371/journal.pone.0316004

**Published:** 2024-12-13

**Authors:** Benedict Weobong, Franklin N. Glozah, Hannah B. Taylor-Abdulai, Eric Koka, Nancy Addae, Stanley Alor, Kid Kohl, Prerna Banati, Philip B. Adongo, David A. Ross

There are errors in the author affiliations. The correct affiliations are as follows:

Benedict Weobong^1,2^, Franklin N. Glozah^2^, Hannah B. Taylor-Abdulai^3^, Eric Koka^4^, Nancy Addae^4^, Stanley Alor^2^, Kid Kohl^5^, Prerna Banati^6^, Philip B. Adongo^2^, David A. Ross^7^

**1** School of Global Health, Faculty of Health, York University, Toronto, Canada, **2** Department of Social and Behavioural Sciences, School of Public Health, University of Ghana, Accra, Ghana, **3** Department of Physician Assistant Studies, University of Cape Coast, Cape Coast, Ghana, **4** Department of Sociology and Anthropology, University of Cape Coast, Cape Coast, Ghana, **5** Technical Advice and Partnerships Department, The Global Fund, Geneva, Switzerland, **6** Adolescent and Young Adult Health Unit, Maternal, Newborn, Child and Adolescent Health and Ageing Department, World Health Organization, Geneva, Switzerland, **7** Institute for Lifecourse Health Research, Stellenbosch University, Stellenbosch, South Africa.
