# Supplementary material for: Reaching adolescents with health services: Systematic development of an adolescent health check-ups and wellbeing programme in Ghana (Y-Check, Ghana)
Source: PLoS One. 2024 Aug 27;19(8):e0304465. doi: 10.1371/journal.pone.0304465 (PMC11349232; doi:10.1371/journal.pone.0304465)
Supplement: S1 Appendix — (PDF) [file pone.0304465.s001.pdf]

S1 Appendix

Table A: Key informant information

Category	Sex		
	**Male**	**Female**	**Total**
Head teachers	1	2	3
School Health Education Programme Coordinator, Cape Coast Metropolis	0	1	1
Health workers	4	7	11
NGO	1	0	1
**Total**	6	10	16

Table B: Number of adolescents who were registered in each school year in Cape Coast (2016/2017) academic year

	Public			Private			Combined		
	Male	Female	Sub-total	Male	Female	Sub-total	Male	Female	Total
Primary 1	1210	1,259	2,469	1,073	1,019	2,092	2,283	2,278	4,561
Primary 2	1,394	1,359	2,753	1,029	974	2,003	2,423	2,333	4,756
Primary 3	1,397	1,472	2,869	961	944	1,905	2,358	2,416	4,774
Primary 4	1,442	1,557	2,999	863	887	1,750	2,305	2,444	4,749
Primary 5	1,348	1,453	2,801	767	846	1,613	2,115	2,299	4,414
Primary 6	1,247	1,361	2,608	711	693	1,404	1,958	2,054	4,012
Primary Sub-total	8,038	8,461	16,499	5,404	5,363	10,767	13,442	13,824	27,266
JHS 1	1,395	1,414	2,809	641	676	1,317	2,036	2,090	4,126
JHS 2	1,233	1,307	2,540	565	606	1,171	1,798	1,913	3,711
JHS 3	1,138	1,125	2,263	587	546	1,133	1,725	1,671	3,396
[a]JHS Sub-total	3,766	3,846	7,612	1,793	1,828	3,621	5,559	5,674	11,233
SHS 1	3,156	2,393	5,549	104	73	177	3,260	2,466	5,726
SHS 2	3,928	2,570	6,498	33	35	68	3,961	2,605	6,566
SHS 3	3,720	2,313	6,033	36	46	82	3,756	2,359	6,115
[b]SHS Sub-total	10,804	7,276	18,080	173	154	327	10977	7430	18,407
Overall Total	22,608	19,583	42,191	7,370	7,345	14,715	29,978	26,928	56,906

[a]JHS: Junior High School; [b]SHS: Senior High School

Table C: Number of adolescents who were registered in each school year in Cape Coast (2017/2018 academic year)

2017/2018	Public			Private			Combined		
	**Male**	**Female**	**Sub-total**	**Male**	**Female**	**Sub-total**	**Male**	**Female**	**Total**
Primary 1	1210	1,259	2,469	1,073	1,019	2,092	2,283	2,278	4,561
Primary 2	1,394	1,359	2,753	1,029	974	2,003	2,423	2,333	4,756
Primary 3	1,397	1,472	2,869	961	944	1,905	2,358	2,416	4,774
Primary 4	1,442	1,557	2,999	863	887	1,750	2,305	2,444	4,749
Primary 5	1,348	1,453	2,801	767	846	1,613	2,115	2,299	4,414
Primary 6	1,247	1,361	2,608	711	693	1,404	1,958	2,054	4,012
**Primary Sub-total**	**8,038**	**8,461**	**16,499**	**5,404**	**5,363**	**10,767**	**13,442**	**13,824**	**27,266**
JHS 1	1,395	1,414	2,809	641	676	1,317	2,036	2,090	4,126
JHS 2	1,233	1,307	2,540	565	606	1,171	1,798	1,913	3,711
JHS 3	1,138	1,125	2,263	587	546	1,133	1,725	1,671	3,396
[a]**JHS Sub-total**	**3,766**	**3,846**	**7,612**	**1,793**	**1,828**	**3,621**	**5,559**	**5,674**	**11,233**
SHS 1	3,346	2,681	**6,027**	**-**	**-**	**-**	3,346	2,681	6,027
SHS 2	3,046	2,462	**5,508**	**-**	**-**		3,046	2,462	5,508
SHS 3	3,827	2,404	**6,231**	**-**	**-**	**-**	3,827	2,404	6,231
[b]**SHS Sub-total**	**10,219**	**7,547**	**17,766**	**-**	**-**	**-**	**10,219**	**7,547**	**17,766**
**Overall Total**	**22,023**	**19,854**	**41,877**	**7,197**	**7,191**	**14,388**	**29,220**	**27,045**	**56,265**

[a]JHS: Junior High School; [b]SHS: Senior High School

Table D: Number of adolescents who were registered in each school year in Cape Coast (2018/2019 academic year)

2018-2019	Public			Private			Total		
	**Male**	**Female**	**Sub-total**	**Male**	**Female**	**Sub-total**	**Male**	**Female**	**G-Total**
Primary 1	1,118	1,171	2,289	1,231	1,213	2,444	2,349	2,384	4,733
Primary 2	1,195	1,228	2,423	1,187	1,120	2,307	2,382	2,348	4,730
Primary 3	1,386	1,343	2,729	1,057	1,030	2,087	2,443	2,373	4,816
Primary 4	1,446	1,430	2,876	1,033	976	2,009	2,479	2,406	4,885
Primary 5	1,301	1,480	2,781	915	914	1,829	2,216	2,394	4,610
Primary 6	1,367	1,459	2,826	885	818	1,703	2,252	2,277	4,529
**Primary total**	**7,813**	**8,111**	**15,924**	**6,308**	**6,071**	**12,379**	**14,121**	**14,182**	**28,303**
[a]JHS 1	1,523	1,636	3,159	538	621	1,159	2,061	2,257	4,318
JHS 2	1,414	1,455	2,869	460	536	996	1,874	1,991	3,865
JHS 3	1,209	1,305	2,514	439	452	891	1,648	1,757	3,405
**JHS total**	**4,146**	**4,396**	**8,542**	**1,437**	**1,609**	**3,046**	**5,583**	**6,005**	**11,588**
[b]SHS 1	5,355	3,553	8,908	-	-	-	5,355	3,553	8,908
SHS 2	3,243	2,884	6,127	-	-	-	3,243	2,884	6,127
SHS 3	2,962	2,428	5,390	-	-	-	2,962	2,428	5,390
**SHS total**	**11,560**	**8,865**	**20,425**	-	-	-	**11,560**	**8,865**	**20,425**
**Total**	**23,519**	**21372**	**44,891**	**7745**	**7680**	**15425**	**31264**	**29052**	**60316**

[a]JHS: Junior High School; [b]SHS: Senior High School
