# Supplementary material for: Reaching adolescents with health services: Systematic development of an adolescent health check-ups and wellbeing programme in Ghana (Y-Check, Ghana)
Source: PLoS One. 2024 Aug 27;19(8):e0304465. doi: 10.1371/journal.pone.0304465 (PMC11349232; doi:10.1371/journal.pone.0304465)
Supplement: Attachment — Submitted filename: Response to reviewers_PONE-D-23-21967.docx [file pone.0304465.s002.docx]

Dear Prof Chenette,

Manuscript: PONE-D-23-21967 Reaching adolescents with health services: systematic development of an adolescent health check-ups programme in Ghana (Y-Check, Ghana)

Thank you for your comments regarding our manuscript and the invitation to revise and resubmit. Please see below a table summarising the response to the concerns raised by the reviewers. All the revisions in the revised manuscript are in track changes or highlighted.

| **Comment/concern** | **Response/action** |
| --- | --- |
| Journal requirements |  |
| 1. Please ensure that your manuscript meets PLOS ONE's style requirements, including those for file naming. | The manuscript has been formatted to meet PLOS ONE's style requirements, including those for file naming. |
| 2. We note that the grant information you provided in the ‘Funding Information’ and ‘Financial Disclosure’ sections do not match.   When you resubmit, please ensure that you provide the correct grant numbers for the awards you received for your study in the ‘Funding Information’ section. | Thank you. We have now ensured the grant information is aligned in the two sections. |
| 3. We note that you have stated that you will provide repository information for your data at acceptance. Should your manuscript be accepted for publication, we will hold it until you provide the relevant accession numbers or DOIs necessary to access your data. If you wish to make changes to your Data Availability statement, please describe these changes in your cover letter and we will update your Data Availability statement to reflect the information you provide. | We wish to amend our data availability statement. We have described these changes in our cover letter. |
| 4. Please amend your list of authors on the manuscript to ensure that each author is linked to an affiliation. Authors’ affiliations should reflect the institution where the work was done (if authors moved subsequently, you can also list the new affiliation stating “current affiliation:….” as necessary). | The list of authors and author affiliations have been amended to conform with the journals’ requirements. |
| 5. We note that you have referenced (unpublished) on page 9, which has currently not yet been accepted for publication. Please remove this from your References and amend this to state in the body of your manuscript: (ie “Bewick et al. [Unpublished]”) | We have checked and can confirm this reference is not in our manuscript. |
| 6. Please include your full ethics statement in the ‘Methods’ section of your manuscript file. In your statement, please include the full name of the IRB or ethics committee who approved or waived your study, as well as whether or not you obtained informed written or verbal consent. If consent was waived for your study, please include this information in your statement as well. | A full ethics statement including the full name of the IRB that approved the study protocol has been provided in the Methods section. |
| 7. Please include captions for your Supporting Information files at the end of your manuscript, and update any in-text citations to match accordingly. | Thank you. We have now provided this information as requested. |
| Reviewer 1 |  |
| 8. This is a huge study that covered different locations in Cape coast, Ghana. For clarity, the study setting should be separated from the design. This will enhance the appreciation of the uniqueness and content of the design. | The study design as been separated from the study setting. In addition, information about participants has been moved here from the results section. |
| 9. Details about the data analysis was inadequate despite the huge data used in this research. For example, for ‘Identification of key policies, programmes, and interventions‘ step, how were the analysis of the identified policies, programs and interventions carried out? 10. The same applies to ‘Understanding the school health system and school attendance’ step. How were the data and literature identified reviewed and how was school attendance and access evaluated? | These two sections are part of the description of the study design, so how they were analyzed is not included in these sections. We have expanded the data analysis section to include how these were analyzed. |
| 11. The presentation of the results is not following the sequence of the steps in the design. Presenting the results in the logical flow of the steps in the design will make the reading easier. For example, the third step in the design was to identify common adolescent health problems but this was the first result to be presented. It is confusing. | The results section has been reorganised to ensure that it aligns and there is a logical flow of the steps as outlined in the research design section. |
| 12. It appears that the data for identification of the adolescent health problems were not analyzed. What were presented in tables 1 and 2 appears like data from a single study/document (which was not referenced in this section where it was mentioned). The data from the literature search and the key informant interviews should be correctly analyzed and presented clearly to answer this objective. The method of analysis should also be clearly spelt out. | The data presented in Tables 1 and 2 (now Tables 3 and 4) are secondary data from the Global Burden of Disease study. These were analysed to estimate the DALYs for top 10 diseases of concern among adolescents in Ghana using the national data. This has been explained and the source of this data has been presented.  Also, the data analysis section has been revised to highlight how the data from the literature search and the key informant interviews were analysed. |
| 13. The description of the study settlements, school characteristics (lines 329-332) presented should have been under study settings. They are not part of the results. | Information about the study setting which was in the results section have been moved to the study setting section in the Methods. |
| 14. The description of the participants in lines 282-285 is difficult to comprehend. It should be made clear. | The description of participants has been revised to make it clearer. |
| 15. Please, provide a reference for the definition of ‘adolescent friendly spaces’ (lines 479-481). It appears that the only question that adolescents ask there is only about sex. This is incorrect and misleading. | A reference has been provided for ‘adolescent friendly spaces’ and its definition has been modified to indicate that it focuses on adolescent health but prioritises or is biased towards sexual health. |
| 16. The demographic characteristics of the Community Advisory Board should be added for better understanding of their contributions | A brief description of the composition of the Community Advisory Board has been provided. |
| 17. Lines 361-363- This is discussion and should be deleted. All other discussions (e.g. Lines 389-401, 365-366) in this section should be transferred to discussion section. | Thank you for your suggestion. We have now discussed the implications of the data on school attendance in the discussion section.  “Another key learning is the importance of thinking through issues of yield in terms of numbers available to screen and follow-ups. In our study we carefully examined data on school attendance and the transition from basic school to senior high school (SHS). Our study setting Cape Coast is particularly unusual in terms of SHS education because it has some of the best SHSs in Ghana and thus attracts many (usually affluent) students from outside the Metropolitan area of Cape Coast. This phenomenon presents some challenges in ensuring adolescents are able to be followed up after screening”  Consequently, we have deleted the text in the results section. |
| 18. Some sentences are not clear- lines 223-226, 282-285 19. Some sentences are also too long making comprehension difficult e.g., lines 239-245 20. There was a mixture of British and American English. Please, stick to one. | These sentences have been revised and made concise and clear.  We have revised the paper to ensure that we have used British English throughout the paper. |
| 21. What does Y mean in Table 6? This should be defined. 22. Mid upper arm circumference is used for screening for malnutrition in early childhood. It has no place in adolescence. | Y = Yes, N = No. This has been indicated under Table 6.  Regarding application of MUAC, we respectfully disagree that it has no place in adolescence. MUAC is recommended by WHO for use in screening for thinness among adolescents[1]. |

I look forward to your reply in due course.

Sincerely,

Benedict Weobong

[1].	WHO. IMAI District Clinician Manual: Hospital Care for Adolescents and Adults. Guidelines for the Management of Illnesses with Limited Resources. Geneva2011. p. 760-.
