# Supplementary material for: Reaching adolescents with health services: Systematic development of an adolescent health check-ups and wellbeing programme in Ghana (Y-Check, Ghana)
Source: PLoS One. 2024 Aug 27;19(8):e0304465. doi: 10.1371/journal.pone.0304465 (PMC11349232; doi:10.1371/journal.pone.0304465)
Supplement: Attachment — Submitted filename: Response to reviewers_PONE-D-23-21967_feb24.docx [file pone.0304465.s003.docx]

Dear Prof Chenette,

Manuscript: PONE-D-23-21967R1 Reaching adolescents with health services: systematic development of an adolescent health check-ups programme in Ghana (Y-Check, Ghana)

Thank you for your comments regarding our manuscript and the invitation to revise and resubmit. Please see below a table summarising the response to the concerns raised by the reviewers. All the revisions in the revised manuscript are in track changes or highlighted.

| **Comment/concern** | **Response/action** |
| --- | --- |
| Reviewer 1 |  |
| 1. On one of your diagrams you clearly showed how the study involved three phases which included; situation analysis, feasibility & acceptibility assessments with participants and co-development of an intervention. **However, when the methodology & results are read there is lack of coherence in terms of that illustration.** | Thank you for your comment. We have checked for alignment between the study flow diagram (figure 1), the accompanying text in the methods section, and the results. We are able to confirm that the first phase involved identifying key contextual information, the second focussed on eliciting components of feasibility and acceptability of the proposed routine health check-ups intervention, and the final phase sought to assemble information from phases 1 and 2 into the evolving intervention framework/YCheck prototype. Each of these phases involved multiple steps and these were clearly presented.  The results followed the same logical sequence: ***Stage 1: Key contextual information identified; Stage 2: Potential health check-ups (Y-Check) components identified; Stage 3: Health check-ups (Y-Check) framework formulated*** |
| 2. There are methodological gaps on who exactly the "key informants" comprised of? | Thank you for this comment and we apologise for not making this clear. We have now added text to clarify this in the manuscript. This is what we added: “Key informants comprised a cross-section of stakeholders including: key staff of Ghana Health Service; Ghana Education Service; leaders of relevant NGOs and community-based organizations working on adolescent health; younger adolescents, older adolescents, and parents of adolescents”. (lines 116 to 119) |
| 3. Lack of clarity on search strategy of undertaking literature review? How did you analyze it? | Thank you for your comment. We have now provided information on the search strategy (lines 159 to 173). We have also elaborated a bit more on our analysis of the rapid literature review:  *“For the rapid literature review, data was extracted using a standardized template. The data was synthesised using thematic analysis and reported as either ranking, prioritisation, estimation, or observation of trends”* |
| 4. What tools did you use for assessing feasibility and acceptability? | Thank you for your comment, and we apologise if this was not clear in the manuscript. We have now provided the following text in the manuscript to explain how these were assessed.  *“Feasibility and acceptability were qualitatively assessed using two main data sources: the key informant interviews with health services/education staff and adolescents and their parents. The final determination of these critical implementation outcomes was arrived at during the consensus building workshop”*  We have now included this text in the analysis section (lines 273 to 277) |
| 5. There is need for more details in the steps you took in your framework analysis, how did you ensure trustworthiness? 6. In summary, you need to be clear, what data you collected, tools for collecting that data, what information that data provided and how did you analyse it. This all needs to be aligned with your three phrases. | Thank you for your comment. We have now provided some detail in the analysis to address this important observation (lines 278 to 284). We introduced this text:  *“To ensure the reliability and validity of the findings, two researchers first read and familiarized themselves with the data. Following this, few randomly selected interviews were read and initial codes generated through coding parcels of data in a systematic fashion. Based on the coded data and the original research questions, the researchers defined and collated codes into potential themes and finalized the code-book. The two researchers then coded the entire data set. Inter-rater reliability was tested by double coding ten randomly selected interview transcripts. For the rapid literature review, data was extracted using a standardized template”*  We also agree with you that it’s important to be clear in our reporting, and your feedback has helped us to check this again. We are happy to confirm that:   1. we did provide detail on what data was collected in each of the phases (secondary and primary qualitative), 2. we discussed the tools for data collection (literature review data extraction sheets, interview/workshop guides, participatory learning and action (PLA) techniques such as free listing, ranking (1) and visualisation in participatory processes; and 3. we shared our analysis strategy for each major phase of the study. |
| 7. Lastly your results are too detailed as you have incorporated elements of discussions in them. That needs to be ironed out | Thank you for this observation. We agree our manuscript may not be laid out in the typical format, and we incorporated a fair amount of discussion in the results. Given the complex design of our heavily qualitative study, and what we sought to share with the scientific fraternity in terms of a systematic approach to developing an adolescent health check-ups programme, we thought it would be useful to provide a bit of context to some of the results we obtained in each phase and step (other than presenting tables with lots of data that may be challenging to retain for a later discussion. |

I look forward to your reply in due course.

Sincerely,

Benedict Weobong

1.	Chambers R. Participatory Workshops: A Sourcebook of 21 Sets of Ideas and Activities.: Routledge; 2002.
